# Practical Treatise on Obstetrics

**Published:** 1887-07

**Authors:** 


A Practical Treatise on Obstetrics. Vol. IV. Obstetric Operations. The Pathology of the Puerperium. By A. Charpentier, M. D., Paris. Illustrated with lithographic plates and wood engravings. This is also Vol. IV. of the "*Cyclopedia of Obstetrics and Gynecology*" (12 vols.), issued monthly during 1887. Price of the set, $16.50. New York: William Wood & Company.

This book forms the concluding volume of Charpentier's Treatise on Obstetrics, a work which, for breadth of conception and completeness of execution, has no parallel in the whole range of obstetrical literature.

The volume before us treats of obstetric operations and the pathology of the puerperium. Under the former head the author discusses version, the use of forceps, the induction of artificial labor, Cæsarian section, Porro's operation and embryotomy; under the latter puerperal fever is alone included.

The great value of this work consists in the minuteness of detail with which the various obstetrical operations are portrayed. In describing the use of the forceps, for instance, all the presentations and positions to which forceps delivery is applicable are taken up *seriatim*, and directions are given for each. This feature renders the work a valuable one to the general practitioner, who has not had the opportunity to perfect himself in such details by long experience or by clinical instruction.

Charpentier is a strong advocate of the timely use of the forceps, regarding it, justly we think, as a life-saving instrument, devoid of danger, if judiciously and skillfully used before evils have arisen from delay. Between forceps and version he makes the former the operation of choice, the latter the one of necessity, even when the head is movable above the brim. In fact, he does not recognize any circumstances under which version is applicable in vertex presentations above the superior strait. Upon this point we differ with him. Version is a more rapid method of delivery, as a rule, than the forceps in such cases, even in contracted pelves, and is unquestionably safer for the mother. Where forceps cannot be used, version offers a better chance both to mother and child than craniotomy which the author proposes as the alternative to forceps delivery.

In occipito-posterior vertex positions, forcible rotation by means of the forceps is recommended, a procedure which will find few advocates at the present day. Aside from the difficulty and danger of this method, the fact that spontaneous rotation usually occurs as soon as the head reaches the floor of the pelvis is the strongest argument against such a course.

The axis-fraction forceps finds little favor at Charpentier's hands. For making traction in the axis of the superior strait, he contends that Pajot's method of leverage with the classical forceps accomplishes a more perfect axis-traction than Tarnier's instrument. With the head in the cavity of the pelvis he admits that less force is required to effect delivery by the traction rods; yet even this constitutes an objection, since in cases of extreme dystocia the accoucheur will be tempted to use an amount of force which will involve danger to the mother.

In the chapter upon embryotomy a noticeable feature is the slight value which the author places upon the life of the fœtus as compared with that of the mother. While very few will deny that when placed in the balance the latter should always outweigh the former, fewer still would go with Charpentier so far as to say that "the life of the child is not at all comparable to that of the mother, and therefore the accoucheur ought never to hesitate to sacrifice the former in order to increase the chances of the latter."

In the discussion of puerperal fever, the author fully accepts the germ theory of the origin of the diseases included under that name. His treatment, therefore, is essentially antisepsis; yet he does not believe that the extreme antiseptic methods of prophylaxis advocated by Thomas and others are necessary outside of maternities. In normal labors in private practice, strict cleanliness is sufficient, and only when septic infection has actually occurred are antiseptic measures indicated. Strange to say, in spite of his full acceptance of the septic theory of puerperal fever, he condemns the removal from the uterus of retained fragments of placenta, membranes or blood-clots. He says: "We allow such remnants to detach themselves or wait for their appearance at the cervix before attempting their removal."

It is unfortunate that so valuable a work as this should suffer from a poor translation. A translator should be able to write his own language at least correctly, if not elegantly. "To involute," "are being obtained," "had best attend," "the child asphyxiates," "resume," in the sense of to summarize, "to decompose statistics," meaning to analyze, "fatally" for inevitably, "ever" for always, are samples of errors which are met with on nearly every page. Such sentences as the following (page 329) are of frequent occurrence: "At first with effort, the vomiting gradually merges into pure regurgitation, becomes incessant and in surprising amount." To the lover of pure English the constant repetition of such inaccuracies of language constitutes a serious blemish. Aside from this fault there is very little in the volume to criticize and much to admire.

